# Compositional Shifts and Assembly in Rhizosphere-Associated Fungal Microbiota Throughout the Life Cycle of *Japonica* Rice Under Increased Nitrogen Fertilization

**DOI:** 10.1186/s12284-023-00651-2

**Published:** 2023-08-01

**Authors:** Hangyu Dong, Haoyuan Sun, Conglin Chen, Mingyu Zhang, Dianrong Ma

**Affiliations:** 1 Key Laboratory of Northeast Rice Biology and Breeding, Rice Research Institute, Shenyang Agricultural University, Shenyang, China; 2 Agronomy College, Shenyang Agricultural University, Shenyang, China; 3 Agronomy College, Liaodong University, Dandong, China

**Keywords:** Fungal microbiome, Rhizosphere, Rice, Plant growth stage, Nitrogen fertilization, Soil biochemical properties

## Abstract

Soil fungal microbiomes facilitate a range of beneficial functions for their host plants, and rhizosphere fungal community composition, richness, and diversity affect plant growth and development, and crop yield. Therefore, exploring the community structure and assembly of the rhizosphere fungal microbiome and its relationship with soil biochemical properties are fundamental to elucidating how rice plants benefit from their fungal symbionts. In this study, soil samples were collected at seedling, tillering, heading, and ripening stages of rice subjected to three levels of nitrogen fertilization. Plant growth demonstrates a substantial influence on fungal community composition and diversity. From the tillering to the ripening stage, the fungal communities were governed by homogenizing dispersal and dispersal limitation. The prevalence of Glomeromycota, the beneficial fungi, was considerably higher during the heading stage compared to the three other growth stages. This increase in abundance was strongly associated with increased levels of soil nutrients and enhanced activity of nitrogen acquisition enzymes. This may be a strategy developed by rice grown in flooded soil to recruit beneficial fungi in the rhizosphere to meet high nitrogen demands. Our study findings contribute to elucidating the influence of plant development and nitrogen fertilization on the structure and composition of the fungal community as well as its relationship with soil key soil nutrient content and nitrogen-related enzyme activities. They also illustrate how a shift in the fungal community mediates and reflects the effects of nitrogen fertilization input in rice agroecosystems. These findings provide new insights into the effects of changes in nitrogen application in rice rhizosphere at different growth stages on fungal communities and soil biochemical characteristics.

**Supplementary Information:**

The online version contains supplementary material available at 10.1186/s12284-023-00651-2

## Background

Rice is the primary food source for half of the global population, making its productivity crucial for ensuring food security worldwide (Godfray et al. 2010). Rice yield is influenced by various factors such as climate, water availability, nutrient levels, and biological elements (Kim et al. 2021). Soil microbial communities play a pivotal role in regulating carbon, nitrogen, and other inorganic nutrient cycles, thereby influencing the growth and development of rice (Jansson and Hofmockel 2018). The rhizosphere contains microbial communities that play a vital role in supporting the host plant. These communities provide additional gene pools, effectively acting as the plant's second or extended genome (Berendsen et al. 2012). Plants rely on the mutually beneficial interactions between their roots and soil microbes to improve nutrient availability and stimulate growth (Edwards et al. 2015). Soil microorganisms play a crucial role in converting essential nutrients into more accessible forms for plant absorption (Zhang et al. 2009).

Among these microorganisms, soil fungal communities actively maintain the equilibrium of carbon and nutrients (Žifčáková et al. 2016). Additionally, they facilitate the cycling of phosphorus and nitrogen by transforming organic compounds of phosphorus and nitrogen into mineral forms (Treseder and Lennon 2015), and fungi are more critical than bacteria in the decomposition process and facilitate the interaction between soil and plants (Baldrian et al. 2012; Stursová et al. 2012). Arbuscular mycorrhizal fungi (AMF) are distributed on more than 80% of land plants and help plant roots absorb nutrients from the soil (Zhang et al. 2017). AMF can increase the branch dry weight and grain yield of rice under pot and field conditions (Secilia and Bagyaraj 1994) and AMF inoculation under field conditions increased the nitrogen concentration in shoot tissue (leaves and stems) and grains, and the grain yield of inoculated rice was 14–21% higher than that of non-inoculated rice at the ripening stage (Solaiman and Hirata 1997). Therefore, the role of fungi, including AMF, in flooded rice fields, particularly its impact on the nutritional status of rice has become the focal point of recent research (Zhang et al. 2014).

Edwards et al. (2018) found that the composition of bacterial and archaeal microbiota in the soil undergoes changes throughout the growth cycle of rice plants. However, our understanding of the dynamics of fungal community shifts and their patterns of assembly during the plant's life cycle is limited. To gain deeper insight into microbial ecology, it is crucial to accurately assess the relative impact of deterministic processes, including homogeneous and heterogeneous selection, as well as stochastic processes, such as drift and diffusion constraints, on the overall microbial community, is essential for advancing our knowledge in the field of microbial ecology, as emphasized by Stegen et al. (2012). Recent developments in statistical techniques have facilitated the elucidation of the impact of deterministic and stochastic processes on microbial community structure. Metrics like mean nearest taxon distance (MNTD), nearest taxon index (NTI), beta MNTD (βMNTD, and Beta NTI (βNTI) have been introduced and applied for this purpose (Stegen et al. 2012). These metrics allow for the quantification of phylogenetic turnover within communities from a single sample, employing MNTD and NTI. Conversely, the turnover of phylogenetic composition across different temporal and spatial scales, known as β-phylogenetic diversity, is assessed using βMNTD and βNTI (Stegen et al. 2012). For instance, researchers have employed these methods to investigate and compare the impact of deterministic and stochastic processes on taxonomic and functional microbial community selection in rhizosphere soil (Mendes et al. 2014). These techniques have been proven valuable in elucidating the dynamics underlying governing microbial communities and their responses to various ecological factors. Recent studies have highlighted the significance of environmental changes in shaping the relative contributions of random and deterministic processes in microbial community composition and assembly (Dini-Andreote et al. 2015a, b). For instance, alterations in soil organic matter content influence the relative significance of different assembly processes in shaping the bacterial community in soil. The influence of similar environmental factors on the prevalence of deterministic or stochastic processes in the assembly of fungal communities in rice field soil, particularly within the rhizosphere, remains unclear. Comprehensive elucidation of the roles played by deterministic and stochastic processes can facilitate the connection between microbial community composition and crop yield. If specific microbial communities are identified as crucial for enhancing yield, strategies that promote the proliferation of these specific communities can potentially be implemented on a relatively large scale. By encouraging the growth and activity of these beneficial microbial communities, crop yield may be increased (Zhalnina et al. 2018).

Enzyme reactions are integral to all biochemical transformations occurring in soil (Ruggiero et al. 1996), and microorganisms play a vital role in this process by secreting extracellular enzymes that break down complex organic compounds; the products of these enzyme reactions serve as the primary nutrient source for plants. Soil enzymes serve as indicators of microbial function within the soil system, as they reflect the dynamics of the soil microbial community and nutrient availability. This connection among soil enzymes, microbial activity, and nutrient cycling underscores their significance in elucidating soil health (Nannipieri et al. 2017). Our previous results (Dong et al. 2021) showed that soil enzyme activity varied greatly under different nitrogen application rates. The correlations with fungi were more significant than those with the bacterial community. We therefore aimed to investigate the activities of six enzymes (Additional file 1: Table S1) that play essential roles in the nitrogen cycle and nitrogen acquisition for both plants and microorganisms in the soil. Understanding the structural and assembly dynamics of rhizosphere fungi throughout the life cycle of crop hosts is crucial, given their pivotal role in providing beneficial attributes to the plants. It is equally important to establish connections between the fungal community, soil chemical properties, and enzyme activities in the rice rhizosphere, particularly in response to varying levels of nitrogen fertilization and plant development. This study aimed to achieve three objectives: (1) assess the impact of nitrogen fertilization on fungal community structure during different growth stages of rice; (2) investigate the influence of plant development on the stochastic and deterministic assembly processes of fungal communities under different nitrogen application treatments; and (3) examine the alterations in soil chemical properties and enzyme activities resulting from the combined effects of plant development and nitrogen fertilization. Moreover, this study aimed to establish associations between these biochemical properties and the diversity and composition of the fungal community.

## Materials and Methods

### Pot Experiment and Sample Collection

The Japonica rice cultivar ‘Yanfeng47’ is commonly planted in Liaoning province and exhibits moderate maturity, high and stable yield, remarkable rice quality, and moderate susceptibility to rice blast. It was therefore selected for this study. It has been regularly planted over the last 5 years in our laboratory to serve as experimental material. The pot experiment was conducted at the scientific research base of Shenyang Agricultural University in Liaoning Province, China (123° 34′ E, 41° 49′ N). The study site experiences an average annual temperature of 8.4 °C and receives approximately 715.5 mm of precipitation. For this study, Hydragric Anthrosol (Fan et al. 2020) soil with a pH of 6.6, total nitrogen content of 1.15 g/kg, total phosphorus content of 0.62 g/kg, and total potassium content of 19.5 g/kg was utilized. After screening the soil (removing grass and stones), we mixed it and placed it (approximately 20 kg) in individual pots to a height of 50 cm and at a diameter of 22 cm. The experiment constituted three nitrogen fertilization levels: 120, 180, and 240 kg N/hm^2^ per rice-growing season. Urea, containing 46% nitrogen (N), was applied as the nitrogen fertilizer in this study. Consistent amounts of phosphorus fertilizer (calcium superphosphate) and potassium fertilizer (potassium chloride) were applied across all treatment groups, at a rate of 375 kg/hm^2^ for phosphorus and 112.5 kg/hm^2^ for potassium. In this study, rhizosphere samples were collected at four distinct time points during the rice-growing season: 20th June 2021 (referred to as the seedling stage), 13th July 2021 (tillering stage), 5th August 2021 (heading stage), and 21st September 2021 (ripening stage). Thirty pot replicates were set up for each nitrogen fertilizer treatment, and three rice plants were planted in each pot. Destructive sampling was performed at the tillering, heading, and ripening stages, and nine pots were used at each sampling time. Obtaining rice rhizosphere soil under flooded conditions is impossible. Therefore, watering was stopped 3–5 days before each sampling time, and the pots were covered with plastic film on rainy days to avoid natural precipitation which would affect the sampling process. When there was no water layer in the pot and the soil was moist (soil moisture: 70–80%), rhizosphere soil samples were collected as follows. First, the polyethylene pot was carefully cut using a knife. Next, the rice plant was gently removed from the soil and lightly shaken to dislodge loosely attached soil clumps. The remaining soil attached to the rice roots was meticulously collected by brushing it off. Throughout the sampling process, all instruments used were disinfected to maintain hygiene and prevent cross-contamination. In each sampling period, 27 rice rhizosphere soil samples from 9 pots were evenly divided into three parts, and placed in sterile bags maintained at − 80 °C prior for subsequent tests.

### Determination of Soil Properties

The pH of the soil was analysed using a pH analyser (Mettler Toledo, FP20) by measuring the soil-to-water ratio (without CO_2_) at a ratio of 1:2.5 (v/v). The total nitrogen content in the soil was determined via digestion with sulphuric acid followed by the Kjeldahl method. The soil's organic carbon content was determined using the potassium dichromate oxidation heating method (Bao 2000). For the extraction and analysis of soil NH_4_^+^–N and NO_3_^−^–N, 2 M KCl was used as the extracting solution, and an autodiscrete analyser (Smartchem, AMS/Westco, Rome, Italy) was employed. The microbial biomass was determined through the chloroform fumigation-K_2_SO_4_ extraction method (Bao 2000). In brief, 5 g of soil was weighed into a 100 mL beaker, which was then placed in a vacuum desiccator. At the bottom of the desiccator, another beaker containing 50 mL of NaOH solution and one with ethanol-free chloroform were positioned. After sealing, a vacuum pump was used to pump air until the chloroform boiled and after at least 2 min, the valve was closed and placed in the dark at 25° for 24 h. Concurrently, the same mass of soil was weighed without fumigation and placed in another vacuum desiccator as the control. After fumigation, all the soil was transferred to a 250 mL conical flask, then 25 mL of potassium sulphate solution was added, leached by shaking for 30 min, and then filtered. Carbon and nitrogen content in the leaching solution were directly determined using a carbon–nitrogen analyser (Multi N/C 3100 TOC, Analytik Jena AG, Germany). The microbial biomass was determined and the correction factors for carbon and nitrogen were measured to be 2.2 and 1.85, respectively:

microbial biomass carbon: ω(C) = (Organic carbon content of fumigated soil samples-organic carbon content of unfumigated soil samples) × 2.2

microbial biomass nitrogen: ω(N) = (total nitrogen content of fumigated soil samples-total nitrogen content of unfumigated soil samples) × 1.85.

### Determination of Soil Enzyme Activity

The activity levels of urease (UE), nitrate reductase (NR), protease (PT), 1,4-ß-N-acetylglucosaminidase (NAG), nitrogenase (NITS), and leucine aminopeptidase (LAP) in the soil samples were assessed using the double-antibody sandwich enzyme-linked immunosorbent assay (ELISA) method. For each enzyme, microplates coated with specific purified enzyme antibodies were utilized to prepare solid-phase antibodies. Subsequently, the samples were introduced into the microwells coated with monoclonal antibodies, where they were combined with horseradish peroxidase (HRP)-labelled enzyme antibodies, forming antibody-antigen-enzyme-labelled antibody complexes. Following thorough washing with deionized water, the addition of tetramethylbenzidine (TMB; Jiangsu Meimian Industrial Co., Ltd) initiated the colour development process. The presence of HRP catalysed the conversion of TMB into a blue hue, which subsequently turned yellow upon the addition of acids (sodium acetate and citric acid). The spectrophotometric measurement was performed at a wavelength of 450 nm to determine the resulting colour change. The intensity of the colour directly corresponded to the enzyme activity present in the sample. This was determined by comparing the absorbance value of the sample to that of the standard curve.

### DNA Extraction and Amplicon Sequencing

Soil samples were processed to extract total DNA using the PowerSoil DNA Isolation Kit (Mo Bio Laboratories, Inc., Carlsbad, CA, USA) according to the manufacturer's instructions. To assess the purity and concentration of the extracted DNA, electrophoresis was performed on a 1% agarose gel. The ITS region of rRNA genes were then amplified using specific primers, ITS1F/ITS2R (5′-CTTGGTCATTTAGAGGAAGTAA-3′/5′-GCTGCGTTCTTCATCGATGC-3′). PCR amplification was carried out in a 20 µL reaction mixture containing 1 µL of each primer (10 µM), 1 µL of DNA template (approximately 5–50 ng DNA), 0.4 µL of KOD FX Neo (TOYOBO), 10 µL of KOD FX Neo Buffer, and 4 µL of 2 mM dNTPs. The reaction mixture was supplemented with 20 µL of ddH_2_O. The PCR conditions included an initial denaturation step at 95 °C for 5 min, followed by 25 or 30 cycles of 30 s at 95 °C, 30 s at 50 °C, and 1 min at 72 °C. A final extension step was performed at 72 °C for 7 min. The amplified products were analysed using agarose gel electrophoresis, and subsequently purified using AMPure XP beads (Beckman Coulter, Brea, CA, USA) following the manufacturer's instructions. After purification, the PCR products were quantified, homogenized, and prepared as a sequencing library. The library underwent quality checks, and once it met the required criteria, it was sequenced using the Illumina Hiseq 2500 sequencing system (Biomark Biotechnology Co., Ltd., Beijing, China).

### Bioinformatics and Statistical Analysis

The paired-end sequence data obtained from Hiseq sequencing were processed through several steps for quality control and analysis. First, the overlapping regions between the paired-end (PE) reads were identified, and the reads were merged using FLASH v1.2.7 software (http://ccb.jhu.edu/software/FLASH/). The resulting merged sequences represented the original tag data, referred to as Raw Tags. Subsequently, the quality of the merged reads and the effect of the merge were assessed. Trimmomatic v0.33 software (http://www.usadellab.org/cms/?page=trimmomatic) was employed to filter the merged Raw Tags, resulting in high-quality tag data referred to as Clean Tags. To ensure the removal of chimera sequences, UCHIME v4.2 software (http://drive5.com/usearch/manual/uchime_algo.html) was utilized. The chimera sequences were identified and eliminated, resulting in the generation of final effective data known as effective tags. The effective tags were further processed using Usearch software (Edgar 2013). The tags were clustered at a 97% similarity level to form operational taxonomic units (OTUs), which were then taxonomically annotated using the UNITE taxonomy database. The microbial reference database was utilized to compare representative sequences of the Operational Taxonomic Units (OTUs). This comparison facilitated the classification of species corresponding to each OTU, enabling the determination of community composition in the samples at various taxonomic levels such as phylum, class, order, family, genus, and species. The QIIME software (Bolyen et al. 2019) was employed to generate species abundance tables at different taxonomic levels and to analyse beta diversity. Additionally, the alpha diversity indexes of the samples were evaluated using Mothur software (version v.1.30) available at http://mothur.org. The composition of microbial communities is influenced by a range of ecological processes, which encompass both deterministic and stochastic factors. Deterministic processes are driven by ecological selection, while stochastic processes arise from unpredictable disturbances, probabilistic dispersion, and random birth–death events. To evaluate the dynamic interplay between these stochastic and deterministic ecological processes, a randomization approach was employed. A null distribution comprising 999 randomizations was generated for each βMNTD estimate, where βMNTD represents a pairwise distance measure. For each pairwise comparison of the communities, a null βMNTD distribution was created. This analysis allows for the assessment of the relative balance between stochastic and deterministic processes within the microbial ecosystem.

The βNTI is a metric that quantifies the difference between the observed βMNTD and the mean of the null distribution, expressed in standard deviation units. It serves as a tool to assess the impact of random and deterministic processes on microbial community assembly across various temporal and spatial scales. A value of |βNTI|> 2 indicates that the replacement between the two communities is primarily driven by selection. Specifically, βNTI >  + 2 suggests heterogeneous selection, while βNTI < -2 indicates homogeneous selection. Conversely, |βNTI|< 2 suggests that the turnover of the community group is influenced by processes such as homogenizing dispersal and dispersal limitation, implying that the selective environment has a less pronounced role in determining differences (or similarities) between the paired microbial communities.

The analysis of ecological processes was conducted using BMKCloud (www.biocloud.net). For taxonomic resolution of fungal OTUs, FUNGuild v1.0, a flat database available on GitHub (https://github.com/UMNFuN/FUNGuild), was utilized. This database offers a consistent and straightforward method for classifying large sequence libraries into ecologically relevant classes (Nguyen et al. 2016). Statistical analysis was performed using SPSS Statistics for Mac, version 23 (IBM Corp., Armonk, NY, USA). Two-way ANOVA was employed to assess variations in soil nutrient contents and activity levels of soil enzymes across different nitrogen treatments at the tillering and heading stages. Pairwise comparisons were conducted using the least significance difference (LSD) tests (assuming equal variance) or Dunnett's test (assuming unequal variance). Pearson correlation was applied to evaluate the relationships between the mentioned characteristics and fungal communities.

## Results

### Soil Chemical Properties and Microbial Biomass Response to Plant Development, and Nitrogen Fertilization

Soil chemical properties play a crucial role in shaping the soil microbial community.

The tillering and heading stages are crucial for rice production as they are characterized by high nutrient demand and the nitrogen fertilizer utilization directly impacts yield during the ripening stage. To assess this relationship, we measured soil pH, nutrient content, and microbial biomass in the rice rhizosphere at the tillering and heading stages. The corresponding results are presented in Table 1. At the tillering stage, the pH of rice rhizosphere soil initially increased and then decreased, and decreased significantly at the heading stage with the increase of nitrogen application rate. The contents of ammonia nitrogen and nitrate nitrogen under medium nitrogen (N180) and high nitrogen treatment (N240) were significantly (*p* < 0.05) higher than those under the low nitrogen fertilization (N120) treatment. The total nitrogen and organic carbon contents in rice soil increased as the nitrogen fertilization application rate increased. Significant differences were observed between the soil microbial biomass carbon and nitrogen contents at the two growth stages. Specifically, the contents of soil microbial biomass carbon and nitrogen at the heading stage were significantly higher than those at the tillering stage (except for the MBC content under N240 treatment).

**Table 1:** Soil pH and nitrogen nutrients and soil organic carbon and microbial biomass of rhizosphere samples subjected to the three nitrogen fertilization levels at the tillering and heading stages of rice

		pH	NH_4_^+^–N (mg kg^−1^)	NO_3_^−^–N (mg kg^−1^)	TN (g kg^−1^)	SOC (g kg^−1^)	MBN (mg kg^−1^)	MBC (mg kg^−1^)
Tillering	N120	6.08 ± 0.07c	21.8 ± 1.23c	23.5 ± 1.06c	1.31 ± 0.10c	9.9 ± 0.57c	7.2 ± 0.88d	69.2 ± 5.5c
	N180	6.47 ± 0.27b	25.5 ± 2.24b	37.4 ± 1.78a	1.48 ± 0.10b	10.7 ± 0.87bc	8.7 ± 0.18cd	80.2 ± 2.7c
	N240	5.68 ± 0.25d	23.0 ± 1.43bc	34.5 ± 0.52a	1.58 ± 0.08ab	11.7 ± 0.65b	9.9 ± 0.48c	100.0 ± 11.0b
Heading	N120	7.27 ± 0.17a	15.4 ± 1.38d	31.2 ± 1.76b	1.49 ± 0.04b	10.9 ± 0.80bc	19.6 ± 2.06b	105.1 ± 15.0b
	N180	6.27 ± 0.11bc	23.6 ± 0.74bc	36.3 ± 1.70a	1.51 ± 0.03b	11.6 ± 0.22b	21.0 ± 1.63b	168.9 ± 10.6a
	N240	5.55 ± 0.12d	35.2 ± 2.89a	37.3 ± 1.95a	1.70 ± 0.03a	13.0 ± 0.13a	29.7 ± 0.89a	112.6 ± 14.0b

*TN* total nitrogen, *SOC* soil organic carbon, *MBN* microbial biomass nitrogen, *MBC* microbial biomass carbon. Different lowercase letters after one data point indicate significant differences at *p* < 0.05, n = 3

Notably, after the nitrogen fertilization application rate increased from 180 to 240 at the heading stage, the MBC content decreased significantly, while the MBN content increased significantly.

### Nitrogen-Related Soil Enzyme Activity Response to Rice Development and Nitrogen Fertilization

**Fig. 1:**
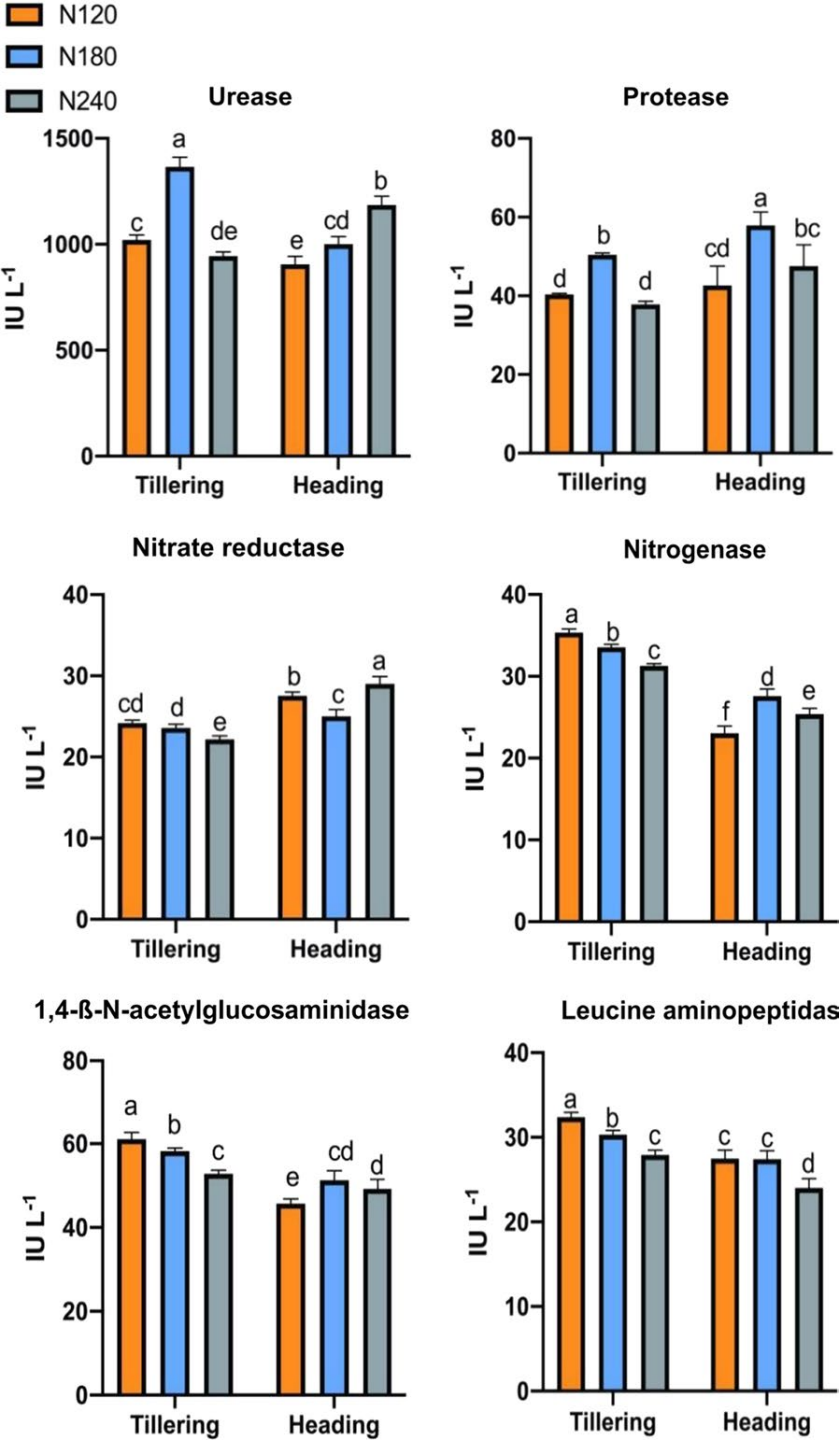
Soil enzyme activities related to nitrogen in the four different nitrogen fertilization levels at the tillering and heading stages. Error bars indicate the standard deviation of three replicates. Different lowercase letters indicate a significant difference between the nitrogen fertilization levels at the two growth stages at *p* < 0.05, n = 3.1 International Unit (IU) = 1 μmol/min

Soil enzymes, which reflect the microbial function in the soil system, integrate the dynamics of soil microbial communities with soil nutrient availability. In this study, we assessed the activities of soil enzymes associated with nitrogen in the rhizosphere soil of rice at the tillering and heading stages (Fig. 1). At the tillering stage, as the nitrogen fertilization application rate increased, soil UE and PT initially increased and then decreased. At the same time, the activities of NR, NITS, NAG, and LAP decreased significantly (*p* < 0.05). When the rice was at the heading stage, the activities of soil PT, NITS, and NAG increased first and then decreased. The UE activity increased significantly as the nitrogen fertilization application rate increased. NR activity was lowest under the N180 treatment, and the treatment with the most insufficient enzyme activity of LAP was N240.

### Response of Fungal Community Structure to Rice Development and Nitrogen Fertilization

**Fig. 2:**
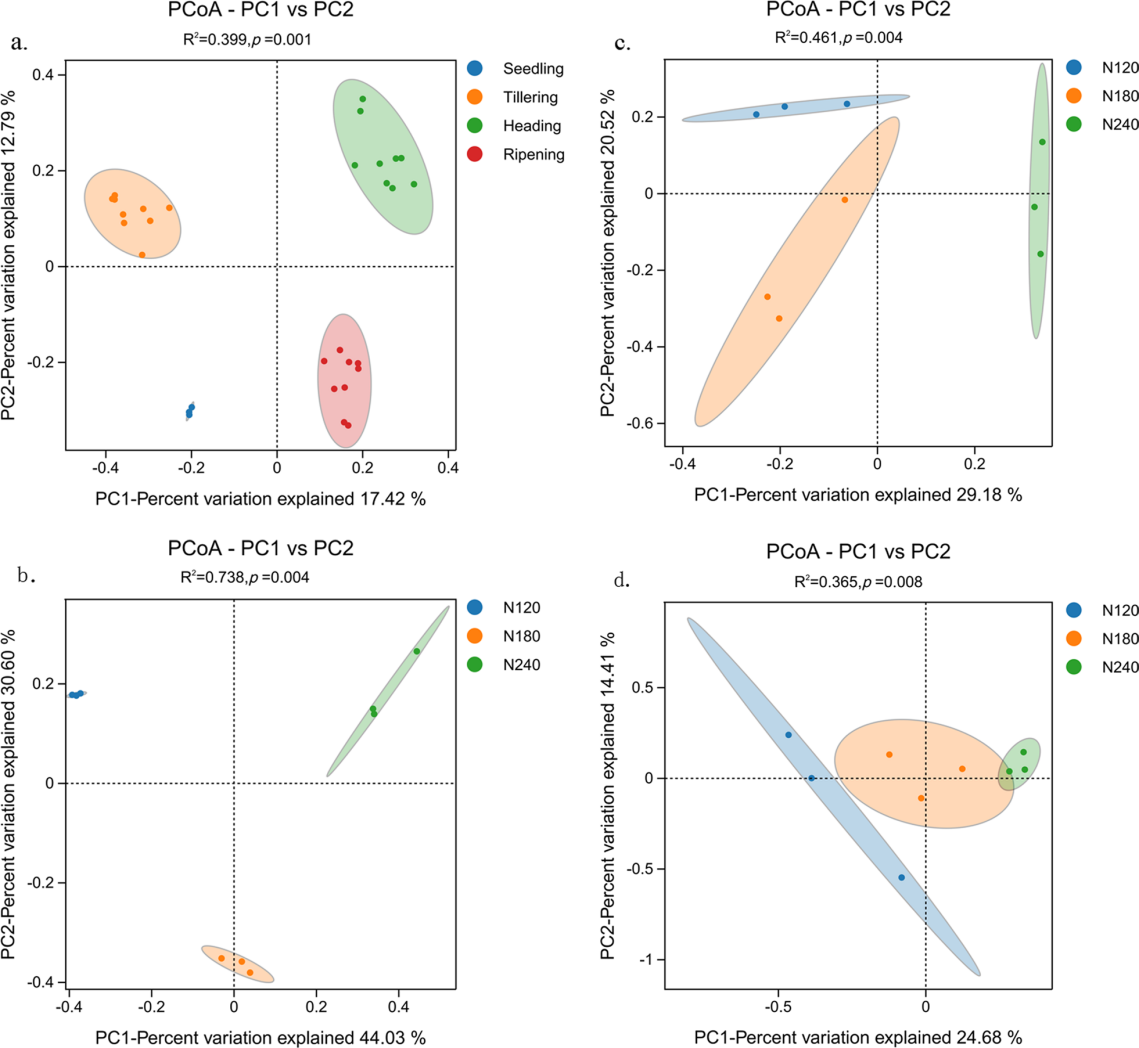

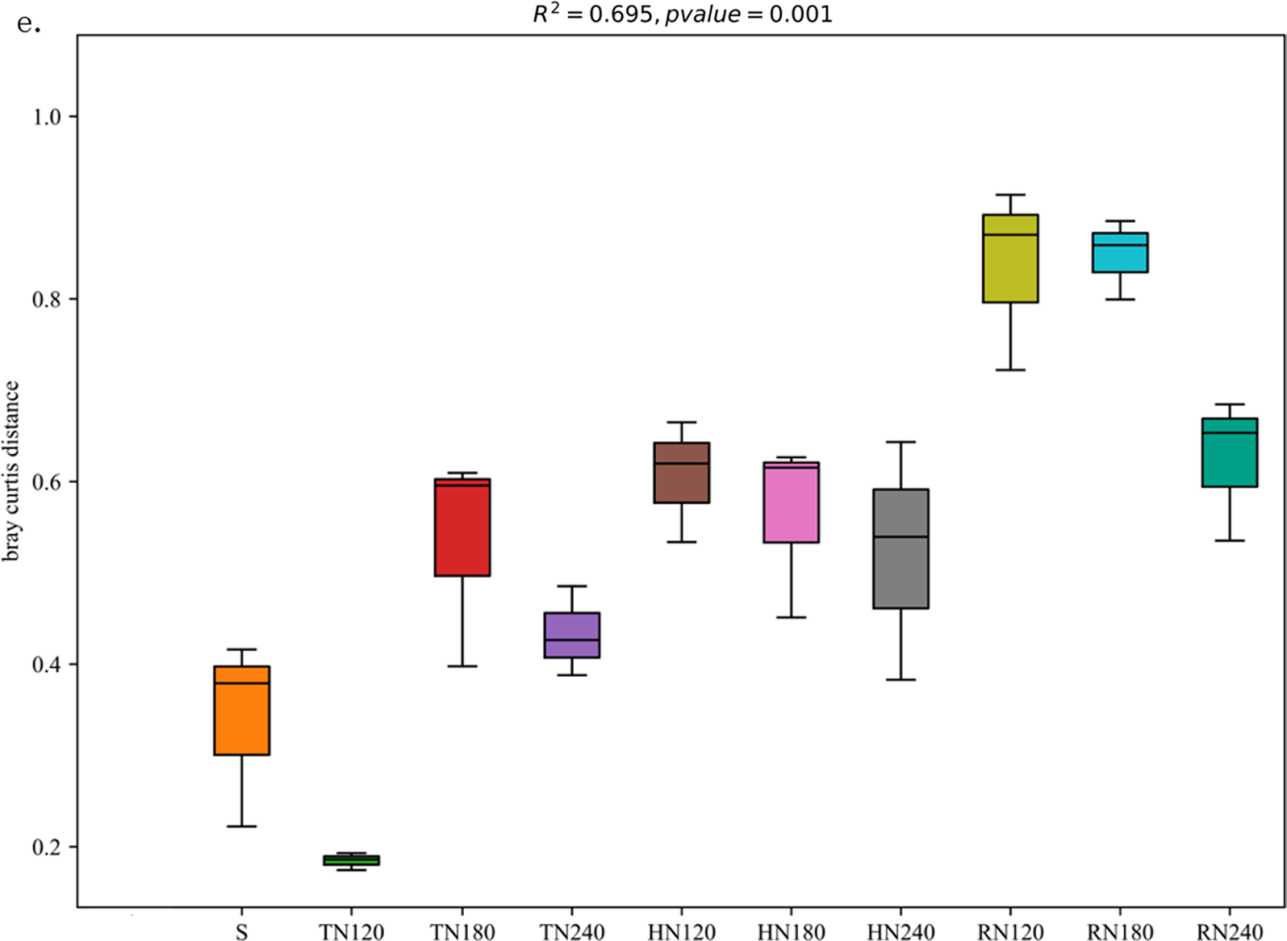
Principal coordinate analysis (PCoA) of the fungal communities in the rhizosphere across the growth stages under the three nitrogen fertilization levels (**a**). And the fungal communities in the rhizosphere at the tillering (**b**), heading (**c**) and ripening (**d**) stages under the three nitrogen fertilization levels. R^2^ value and *p* values calculated by permutational multivariate analysis of variance (PERMANOVA) analysis with 95% confidence level, the confidence ellipse is displayed with a 95% confidence level. Dissimilarity distance **e** showing the differences in fungal community structure across the growth stages in the rhizosphere of rice. *S* seedling stage, *T* tillering stage, *H* heading stage, *R* ripening stage. PCoA and dissimilarity distance were based on Bray–Curtis distance

Principal Coordinate Analysis (PCoA) was conducted at the operational taxonomic unit (OTU) level, as depicted in Fig. 2. The OTUs from soil samples were separated by the rice growth stage, indicating that the growth stage (*p* = 0.01) was the primary underlying cause of the different fungal community structures (Fig. 2a). The effect of nitrogen fertilization on fungal community structure was less than that of plant development. To explore the effect of nitrogen fertilization on fungal community structure during the three growth stages (tillering, heading, and ripening stages), we further conducted PCoA analysis of the soil samples under three nitrogen application treatments in each growth period (Fig. 2b–d). The results show that nitrogen fertilization significantly affected the fungal community structure at the three stages (*p* < 0.05). Further, nitrogen fertilization had the greatest effect on the tillering stage (R^2^ = 0.738, *p* = 0.005), followed by the heading stage (R^2^ = 0.461, *p* = 0.005), and the least effect on the fungal community at the ripening stage (R^2^ = 0.365, *p* = 0.007). The dissimilarity distances among the four growth stages were calculated at each N fertilization level (except for the seedling stage), and the differences in fungal community structure across the growth stages showed an increasing trend from N120 to N180 and decreasing trend from N180 to N240 at the tillering stage. No significant change was observed at the heading stage, but a decreasing trend was observed at the ripening stage with increasing N fertilization level in the rhizosphere of rice.

### Response of Fungal Community Diversity and Richness to Rice Development and Nitrogen Fertilization

**Fig. 3:**
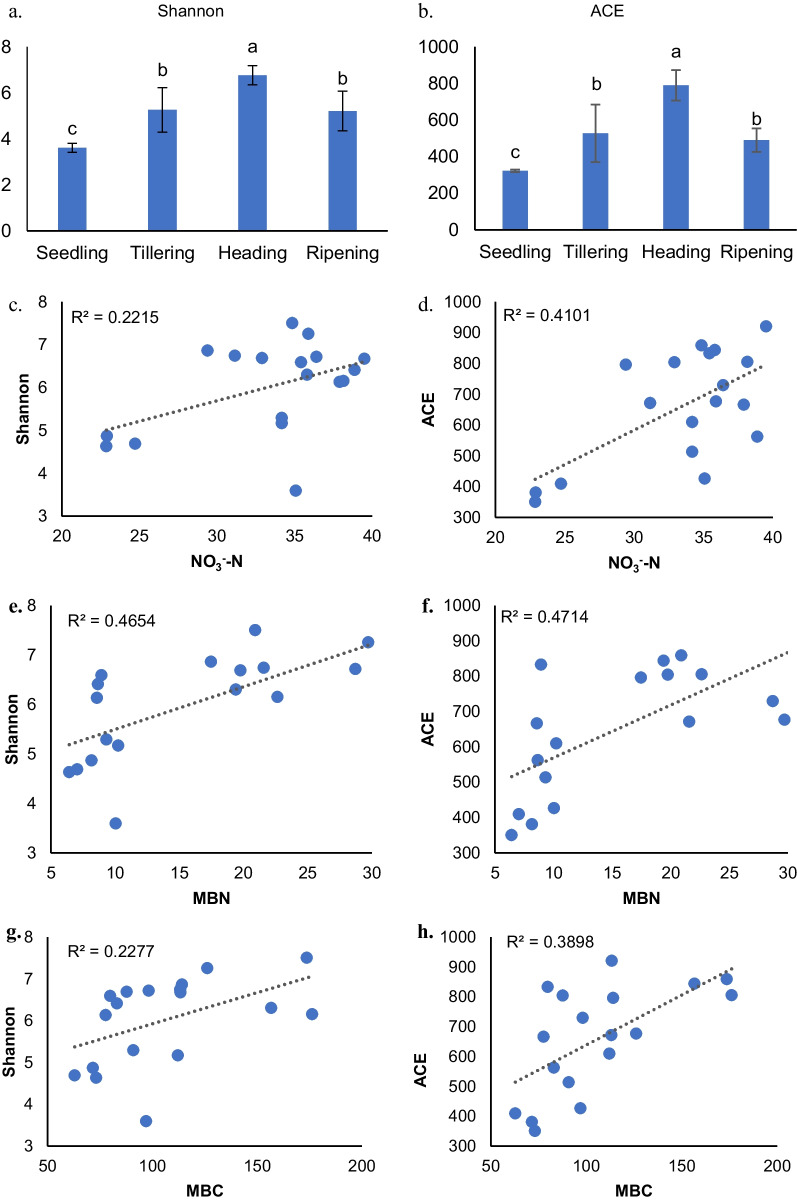
Diversity and richness of fungal communities in the rhizosphere subjected to three levels of nitrogen fertilization at the different growth stages and characterized by Shannon and Ace indices according to Alpha diversity analysis. Different lowercase letters indicate significant difference between the nitrogen fertilization levels at the two growth stages at *p* < 0.05 level

In this research, we utilized the Shannon and Abundance-based Coverage Estimator (ACE) indices to evaluate the fungal species diversity and richness in the rhizosphere soil. The study encompassed three nitrogen fertilization treatments and spanned various growth stages, as depicted in Fig. 3. The results revealed that the fungal community exhibited significantly higher diversity and richness at the heading stage compared to the other three stages. Conversely, the seedling stage exhibited the lowest diversity and richness of fungal communities.

The diversity and richness of the fungal community in the tillering and ripening stages were moderate. The impact of nitrogen fertilization on the diversity and richness of the fungal community in rhizosphere soil showed variations across different stages of plant development (Additional file 1: Fig. S1). The fungal community diversity was highest under the N180 treatment at the tillering and ripening stages. Nitrogen fertilization had no significant effect on the diversity of the fungal community at the heading stage. The richness of the fungal community was not significantly affected by the change in nitrogen application rate at the ripening stages (*p* > 0.05). At the tillering stage, the ACE index of the fungal community was significantly higher (*p* < 0.05) under moderate nitrogen fertilization (N180) compared to low nitrogen (N120) treatment. The results of Spearman correlation analysis demonstrated significant correlations between the Shannon index (Fig. 3c, e, g) and ACE index (Fig. 3d, f, h) of the fungal community with NO_3_^−^–N, MBN, and MBC.

### Fungal Community Composition and Assembly in Response to rice Development and Nitrogen Fertilization

**Fig. 4:**
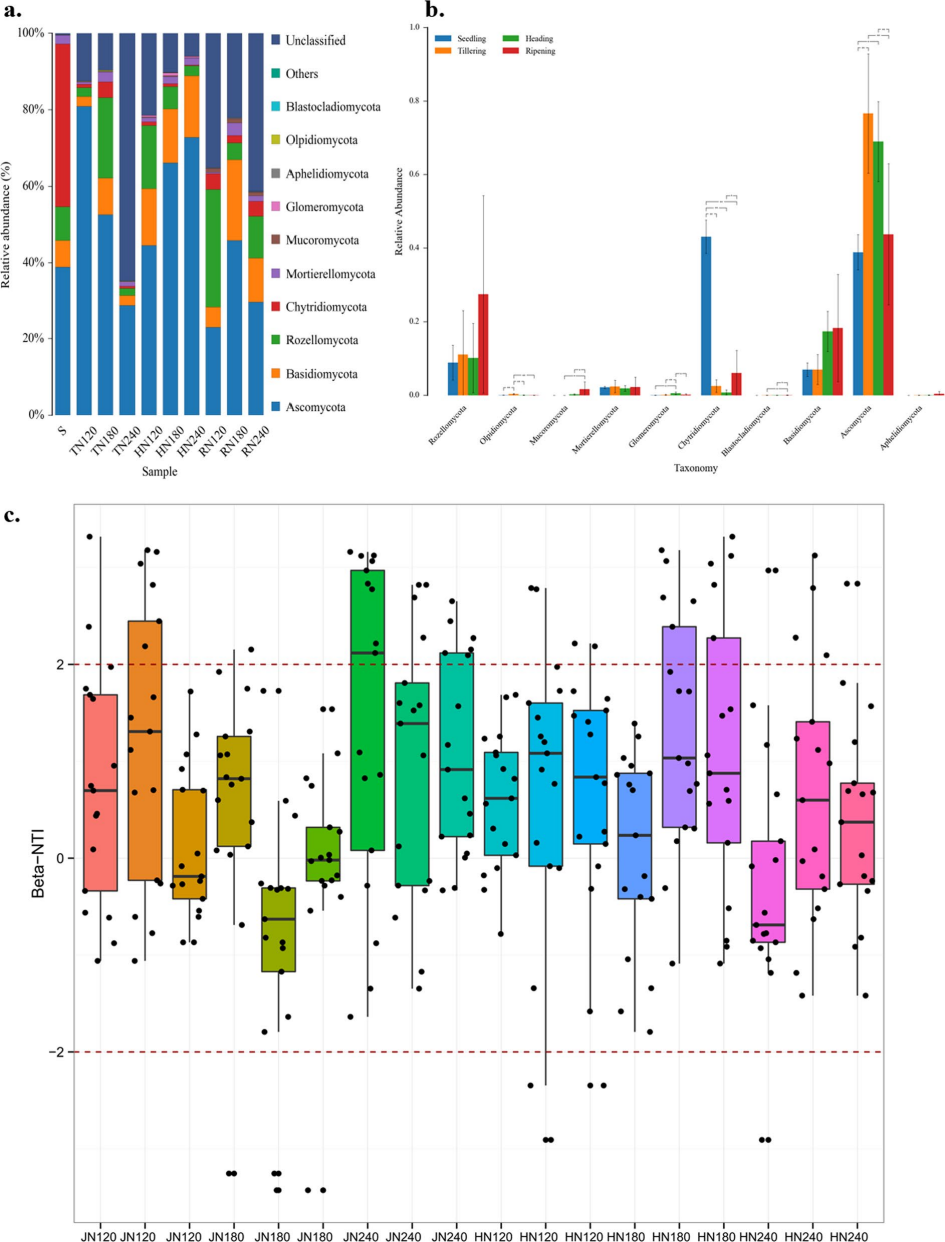

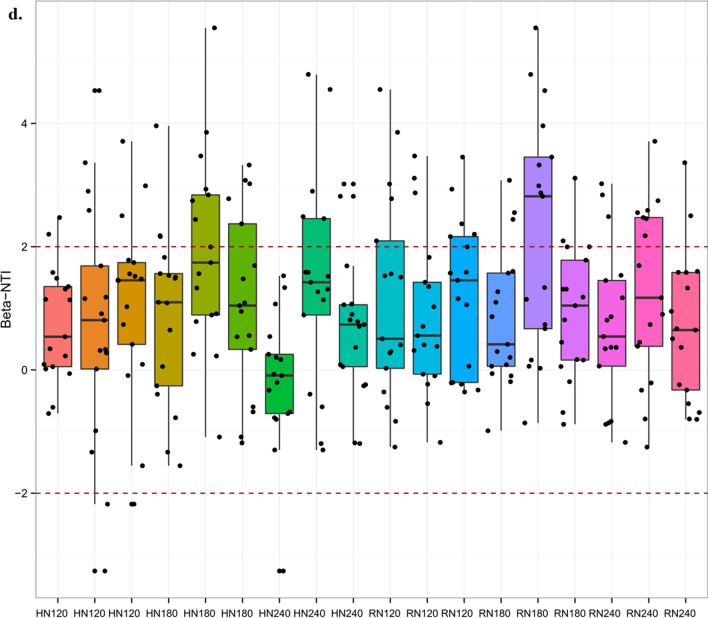
Fungal community composition of the rhizosphere at phylum level (**a**). **b** Significant differences in the fungal community's relative abundance at phylum level based on analysis of variance (ANOVA). β NTI value of the fungal community in the rice rhizosphere from the tillering to heading stage (**c**) and from the heading to ripening stage (**d**)

Samples from rhizosphere soil under different growth stages and nitrogen fertilization levels are shown in Fig. 4. Among the samples, the fungal community composition at the seedling stage was significantly different from that of the other three growth stages (Fig. 4a). Analysis of variance (ANOVA) was used to study the significant difference in the fungal community's relative abundance in soil samples (Fig. 4b). Ascomycota was the dominant fungal community among the four growth stages and exhibited higher relative abundance at the tillering and heading stages. At the seedling stage, the relative abundance of Chytridiomycota was significantly higher than that at the other three stages. As nitrogen fertilization increased, the relative abundance of Ascomycota showed different response patterns at the tillering, heading, and ripening stages. At the tillering stage, it decreased as nitrogen fertilization increased. At the heading stage, it increased as nitrogen fertilization increased. At the ripening stage, it initially increased and then decreased as nitrogen fertilization increased. For Basidiomycota, the relative abundance at the tillering and ripening stages were highest under N180 treatment. In contrast, at the heading stage, the relative abundance of Basidiomycota did not change significantly following an increase in nitrogen fertilization. Compared with a high rate of nitrogen fertilization (N240), Rozellomycota had a greater relative abundance under medium nitrogen (tillering stage) and low nitrogen treatment (heading and ripening stages). During the ripening stage, the relative abundance of Mucoromycota was found to be significantly higher compared to the other three stages. Notably, the relative abundance of arbuscular mycorrhizal fungi (Glomeromycota) was significantly high during the heading stage compared to the other three growth stages. Furthermore, this abundance exhibited an initial increase and subsequent decrease with increasing nitrogen fertilization.

To assess the impact of random and deterministic processes on microbial community assembly across various temporal and spatial scales, we employed β NTI analysis. Specifically, we calculated the β NTI value of the fungal community within the rice rhizosphere at two distinct stages: from the tillering to the heading stage (Fig. 4c), and from the heading stage to the ripening stage (Fig. 4d). To achieve a clearer depiction of the unique characteristics of each sample, diverse colours are utilized to distinguish the replicates that under the same treatment. The results indicate that from the tillering to the heading stage, the fungal communities were governed by homogenizing dispersal and dispersal limitation (79.4%), followed by heterogeneous selection (15.7%), and homogeneous selection (4.9%). From the heading to ripening stage, the fungal communities were governed by homogenizing dispersal and dispersal limitation (72.9%), followed by heterogeneous selection (24.8%) and homogeneous selection (2.3%).

### Function and Phenotype of Fungal Community Responses to Rice Development and Nitrogen Fertilization

**Fig. 5:**
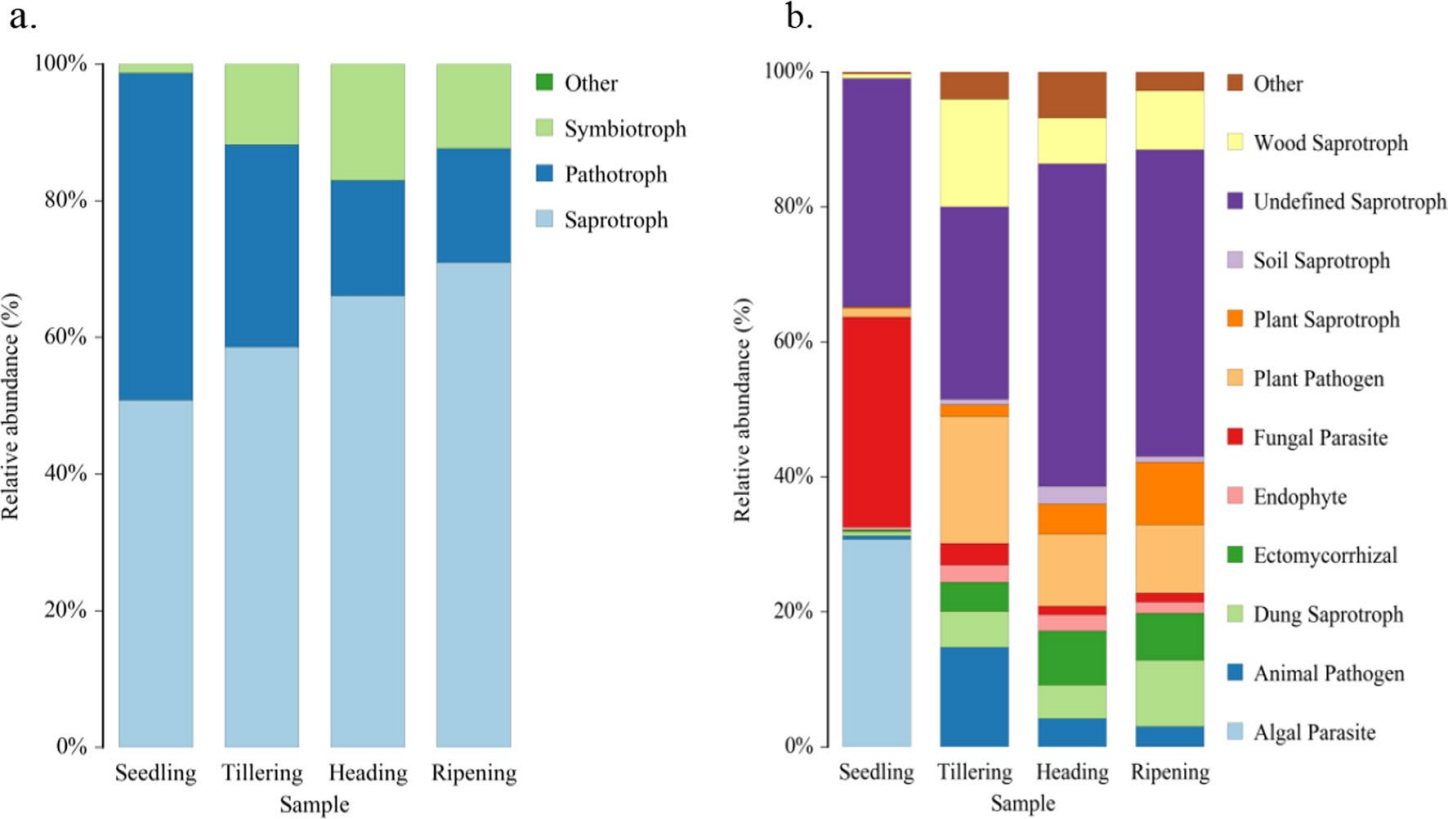
Phenotype (**a**) ecological functions (**b**) of fungal communities were explored using FunGuild

In this study, the phenotype and function of the fungal community at four growth stages were performed using FUNGuild. The analysis results of the fungal phenotype are shown in Fig. 5a. From the rice seedling stage to the ripening stage, the relative abundance of saprotrophs exhibited a noticeable upward trend, while the relative abundance of pathotrophs demonstrated a declining trend from the seedling stage to the heading stage.

There was no significant change in this phenotype from the heading stage to the ripening stage. For symbiotrophs, the relative abundance of this phenotype at the seedling stage was lower than that among the other three growth stages, and the relative abundance of this phenotype peaked at the heading stage.

Figure 5b shows the function of the fungal community during the four growth periods. At the seedling stage, the relative abundance of fungi with the fungal parasite and algal parasite functions was significantly higher than that of the other three stages. In comparison, the relative abundance of other annotated functions was significantly lower than that among the other three stages. At the tillering stage, the relative abundance of fungi with plant pathogen, animal pathogen, and wood saprotrophic functions was the highest. At the heading stage, there was an increase in the relative abundance of fungi with soil saprotroph and ectomycorrhizal functions compared to the other three stages. This finding is consistent with the analysis of Glomeromycota presented in Fig. 2.

### Relationships of Soil Fungal Community, Soil Chemical Properties, and Soil Enzyme Activities

**Fig. 6:**
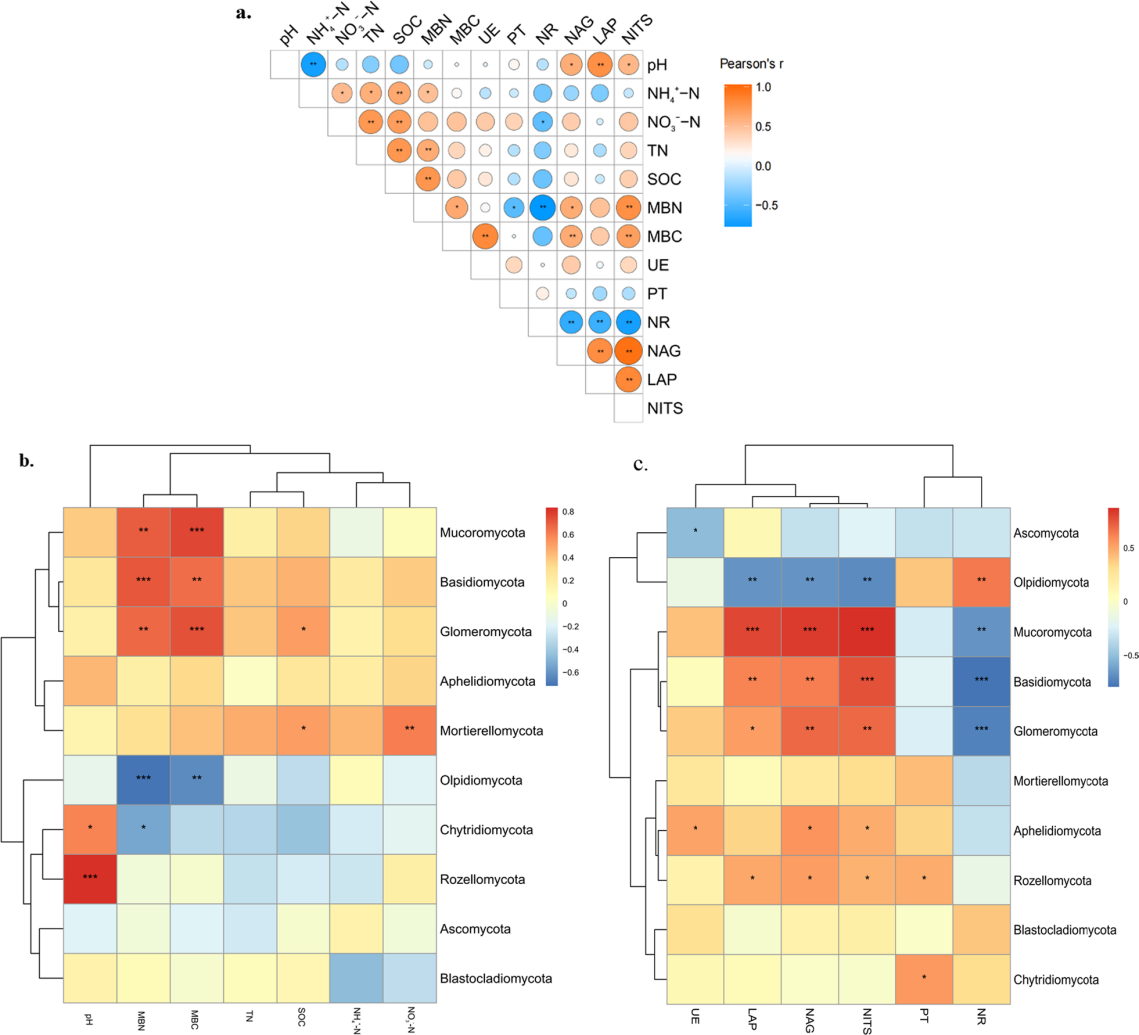
Correlation analysis of soil chemical properties, microbial biomass, and soil enzyme activities (**a**, circle size indicates the strength of the correlation), the relative abundance of soil fungi, soil chemical properties, microbial biomass (**b**), and soil enzyme activities (**c**) in the rhizosphere of rice at the tillering and heading stages. Statistical significance was determined for all pairwise comparisons using Pearson’s method, * indicates significant correlations

To investigate the interrelationships among fungi, nutrients, and enzyme activity, we performed a comprehensive correlation analysis of the relative abundance of soil fungi, soil chemical properties, microbial biomass, and soil enzyme activities within the rice rhizosphere. This analysis specifically focused on the tillering and heading stages (Fig. 6). Our analysis results revealed correlations between several variables (Fig. 6a). Notably, pH exhibited a negative correlation with soil NH_4_^+^–N, TN, and SOC, and the availability of nitrogen, as indicated by NH_4_^+^–N and NO_3_^−^–N, exhibited positive correlations with the levels of soil TN, SOC, MBN, and MBC. These findings highlight the intricate connections between soil parameters and nutrient availability in the rhizosphere of rice at different growth stages. The positive correlation between NH_4_^+^–N and NO_3_^−^–N was significant. The TN and SOC contents were positively correlated with MBN and MBC. As for soil enzymes, the relationship between nitrogen acquisition enzymes (NAG, LAP, and NITS) was significantly positive. The activities of the three enzymes were significantly positively correlated with soil MBN, MBC, and pH. NR activity was negatively correlated with soil nutrients and LAP, NAG, and NITS activities, while UE was positively correlated with soil nutrients and LAP, NAG, NITS, and PT activities.

Spearman correlation analysis was utilized to examine the associations between soil fungal community composition and soil pH as well as nutrient content (Fig. 6b, Additional file 1: Fig. S2a). The results indicated significant positive correlations between the relative abundance of Mucoromycota, Basidiomycota, and Glomeromycota with microbial biomass carbon (MBC) and microbial biomass nitrogen (MBN). Conversely, Oppidomycota displayed significant negative correlations with MBN and MBC content. Soil pH exhibited positive correlation with Rozellomycota and negative correlation with Chytridiomycota. Moreover, the relative abundance of Mortierellomycota showed a significant positive correlation with nitrate nitrogen and organic matter content. Generally, the relative abundances of Mucoromycota, Basidiomycota, Glomeromycota, Aphelidiomycota, and Mortierellomycota were associated with soil pH, ammonium nitrogen, nitrate nitrogen, total nitrogen, organic matter, and microbial biomass.

We observed significant associations between the soil fungal community and soil enzyme activity (Fig. 6c, Additional file 1: Fig. S2b). Specifically, the relative abundance of Mucoromycota, Basidiomycota, and Glomeromycota showed significant positive correlations with the activity of nitrogen acquisition enzymes, including LAP, NAG, and NITS. Conversely, these fungal groups were negatively correlated with NR activity. The relative abundance of Olpidiomycota displayed significant positive correlations with NR activity and negative correlations with LAP, NAG, and NITS activities. UE and PT activities were significantly associated with specific fungal phyla. For instance, UE activity exhibited a positive correlation with the relative abundance of Aphelidiomycota and a negative correlation with the relative abundance of Ascomycota. However, the relative abundance levels of Mortierellomycota and Blastocladiomycota were not significantly correlated with the activity of soil enzymes assessed in this study.

## Discussion

Nitrogen is a primary nutrient that plays a crucial role in determining the growth and productivity of rice crops. Owing to their vital functions in the agricultural ecosystem, including facilitating plant nutrition uptake through the decomposition of soil organic matter (SOM), and their impact on plant health, fungi have gained significant attention. Therefore, conducting a comprehensive study to elucidate fungal response to varying nitrogen fertilizer application rates is imperative. This study aims to investigate the intricate relationships among fungi, soil chemical properties, and soil enzyme activity, which are pivotal in soil nitrogen cycling and plant nitrogen uptake (Phillips et al. 2013). Exploring these dynamics can elucidate the complex interactions in the soil–plant-fungi system and provide valuable insights for optimizing nitrogen fertilizer management strategies in agricultural practices. Our findings revealed that the fungal taxonomic compositions and structures of the rhizosphere communities of rice at different growth stages (seedling, tillering, heading, and ripening stages) and under three nitrogen fertilization application rates (N120, 180, and 240) were assayed by high-throughput ITS rRNA gene fragment sequencing. Soil chemical and microbial biomass were characterized at the tillering and heading stages, and nitrogen-related enzyme activities were investigated using assays.

Plant growth stage was the main driving factor of the rice rhizosphere fungal community structure, followed by the nitrogen fertilizer application rate. This was consistent with previous studies (Breidenbach et al. 2015; Edwards et al. 2018), which stated that the rice development stage can affect the structure of the microbial community. To explore the effect of the nitrogen application rate on the fungal community in the rhizosphere of rice, we performed PCoA analysis on samples at the tillering, heading, and maturity stages, successively. The results showed that the effect of nitrogen application rate on the fungal community structure varied with the growth time of rice. Specifically, the effect of the nitrogen application rate on the structure of the rice rhizosphere fungal community decreased as the plant developed.

The disruption of the fibrous root system during rice transplantation causes mechanical damage to the roots, which are vital for nutrient synthesis, absorption, and storage, thereby affecting aboveground plant parts. The rhizosphere microbiota, closely associated with rice roots, is particularly susceptible to external environmental factors during the root recovery period and our results demonstrated that the seedling and tillering stages exhibited higher relative abundance of pathotrophic fungi compared to the heading and maturity stages (Fig. 5a). To further elucidate the influence of nitrogen application on community structure differences, we conducted PERMANOVA analysis. The results indicated that the R^2^ value of the tillering stage, exceeded those of the heading and seedling stages. This suggests that the application of nitrogen fertilizer had significant impact on the variations in community structure, with the community structure exhibiting relatively high susceptibility to nitrogen application at early growth stage of rice. In addition, the rhizosphere microbial community gradually stabilized and was progressively reduced by the influence of the nitrogen application rate.

The composition of microbial communities is shaped by both deterministic and stochastic processes, as demonstrated in previous studies (Dini-Andreote et al. 2015a, b; Ofiteru et al. 2010). To understand the construction process of the species abundance pattern of rice rhizosphere microorganisms along with spatial and temporal plant development, we used βNTI to evaluate the impact of deterministic and stochastic processes on fungal community assembly under different time–space scales (from the tillering to heading stage, and from the heading to ripening stage). The study results show that the assembly of rice rhizosphere fungal community was mainly driven by stochastic processes (homogenizing dispersal) in the course of the growth period, followed by the deterministic process (heterogeneous selection and homogeneous selection). Kennedy et al. (2005) demonstrated that fungal communities exhibit remarkable resilience to both biotic and abiotic stressors compared to bacterial communities. When environmental selection is attenuated, high diffusion effects lead to low community turnover rates, and a stochastic process drives the community. In contrast with the tillering to heading stage, the degree of fungal community assembly affected by stochastic factors from the heading to ripening stage decreased and the influence of deterministic factors increased. Chen et al. (2021) showed that a random process initially controlled microbial community assembly, and following the initial establishment of the microbial community, deterministic selection may become increasingly critical given the impact of microorganisms on the environment. However, data showing the effect of nitrogen on fungal community assembly in the rhizosphere of rice are lacking. Dini-Andreote et al. (2015a, b) found that deterministic selection increased as nitrogen concentration in soil increased. Our determination of soil chemical properties showed that as nitrogen application increased, the NH_4_^+^–N, NO_3_^−^–N, TN, and SOC contents under N180 and N240 treatment exceeded those under N120 treatment. Therefore, the effect of nitrogen application on soil microbial community assembly may be attributed to the increase in nitrogen concentration.

Fan et al. (2018) revealed that the co-occurrence and assembly process of the diazotrophic community in the wheat rhizosphere were influenced by pH. However, in our study focused on the rhizosphere of rice, we did not observe a significant correlation between soil pH and βNTI values of the fungal community. This discrepancy could potentially be attributed to the limited pH range examined in our study and the specific microbial species detected. Although stochastic processes dominate the assembly of fungal communities in the rice rhizosphere, the role of deterministic progress should not be underestimated owing to the complexity of measuring all environmental variables (Jiao et al. 2016). The structure of the fungal community is predominantly shaped by the concurrent alterations in soil environmental factors (Li et al. 2021). This finding highlights the substantial influence of deterministic processes on the fungal community structure. Therefore, the impact of deterministic processes on the fungal community, particularly in the case of changes in environmental factors, should be considered.

Zeng et al. (2016) found that nitrogen fertilizer affects the diversity of species community by improving the availability of nitrogen in the soil. Rice plant development had more significant impact on the diversity and richness of fungal communities. The lowest levels of species diversity and richness in fungal communities were observed at the seedling stage. This was because the rice at the seedling stage grew in the seedling soil whose type, temperature, and water content among other aspects varied significantly from those of the field soil. After transplanting to the tillering stage, the diversity and richness of fungal communities in the rhizosphere of rice increased significantly. Throughout the rice growth stage, the diversity and richness of the rhizosphere fungal community in the rhizosphere of rice at the heading stage were considerably higher than those in the other three stages. The heading stage is crucial for rice to shift from vegetative to reproductive growth. During this period, the demand for nutrients increases and the interaction between plants and microorganisms becomes robust.

Soil fungi play a crucial role in nutrient cycling as they actively decompose and mineralize soil organic matter, releasing chemically accessible nutrient forms that benefit plants and other microorganisms. This process contributes to the maintenance of the plant-soil biogeochemical cycle (Soong et al. 2018). Saprophytic fungi can effectively degrade stubborn organic matters into unstable substrates (Grinhut et al. 2007). The FUNGuild results confirmed that saprophytic fungi were predominant in soil samples.

In the course of the growth and development of rice, the relative abundance of saprophytic fungi tends to increase, which may be related to plant residues (consisting of polysaccharides derived from cell walls, including cellulose, hemicellulose, and lignin, and pectin) as the primary carbon source of plant saprophytes. In addition, the relative abundance of the pathotrophic fungal community in rice rhizosphere soil was highest at the seedling stage because rice exhibits low resistance to pathogens at the seedling stage. Over the course of the growth and development of the plants, the relative abundance of pathotrophic fungi gradually decreased, indicating that the resistance of plants to soil diseases gradually increased. Our results show that the increase in nitrogen fertilization application significantly influenced the nutrient content, microbial biomass, and soil pH in the rhizosphere of rice. During the tillering and heading stages, when the nitrogen application rate increased from 180 to 240, the soil pH significantly decreased.

Zeng et al. (2016) showed that nitrogen fertilizer directly affected bacterial community and diversity by improving soil nitrogen availability and indirectly affected bacterial composition through changes in soil pH. The present study found that soil NO_3_^−^–N content was significantly positively correlated with fungal community richness. In contrast, pH was not correlated with fungal community diversity and richness, indicating that changes in pH had little effect on the fungal community; compared with bacteria, probably because fungi are less sensitive to the changes in the physical and chemical properties of soil (Jiao et al. 2021).

AMF are important natural and agricultural ecosystems. These fungi can colonize most plant roots and are considered essential factors for promoting nutrient absorption and the growth of host plants (Xiao et al. 2019). In this study, Glomeromycota, the most widespread AMF, was found to be positively correlated with the levels of TN, SOC, NH_4_^+^–N, NO_3_^−^–N, MBN, and MBC. AMF plays critical roles in the soil, including reducing nutrient losses (particularly nitrogen and phosphorus losses) (Köhl and Heijden 2016). Zeng et al. (2021) showed that nitrogen fertilizer has a significant effect on the community composition and structure of soil AMF. The relative abundance of AMF in the rhizosphere of rice at the heading stage was significantly higher than that of the other three growth stages, which may be related to the increased nutrient requirements of rice at the heading stage. Notably, as the nitrogen fertilization application rate increased, the relative abundance of Glomeromycota increased initially (from N120 to N180) and then decreased (from N180 to N240), indicating the negative effect of the high rate of nitrogen fertilizer input on the Glomeromycota in the rhizosphere of rice.

Fungi have a vital role in the dynamics of soil nutrients and can engage in competition with decomposer microbial communities for organic nitrogen (Averill et al. 2014; Phillips et al. 2013). It is noteworthy that various fungal species display diverse abilities when it comes to utilizing nitrogen and phosphorus from different sources (Tedersoo et al. 2012). Consequently, fungi have broader implications for ecosystem-level processes, as alterations in soil fungal community composition can induce changes in soil enzyme activity, ultimately influencing long-term soil nitrogen and other nutrient cycles (Baldrian and Valásková 2008). The application of nitrogen fertilizer had specific effects on the activities of UE, NR, PT, NITS, NAG, and LAP, soil enzymes that are related to the nitrogen cycle. UE hydrolyses urea into water and ammonium nitrogen. At the tillering stage, when the nitrogen application rate was 180 kg, UE activity in the rhizosphere of rice was significantly higher than that in the other two nitrogen treatments, indicating that excessive nitrogen fertilizer application inhibits UE activity in the soil. The response pattern of PT to unreasonable nitrogen fertilizer application is similar to that of UE.

Soil NR is responsible for nitrogen fixation in the soil. NAG and LAP enzymes are involved in nitrogen acquisition processes. NAG is responsible for decomposing N-acetyl-β-D-glucosamine, while LAP catalyses the cleavage of amino acids or proteins, facilitating nitrogen acquisition. These three enzymes are regarded as soil nitrogen acquisition enzymes. We found that their activities decreased significantly during the tillering stage as the nitrogen application rate increased. This may be related to the rise in available nitrogen content in the soil. Correlation analysis shows that the activities of the three nitrogen acquisition enzymes are significantly positively correlated with the relative abundances of Mucoromycota, Basidiomycota, and Glomeromycota, indicating that these three fungi have a positive effect on improving soil enzyme nitrogen acquisition enzyme activities. Members of the Mucoromycota exhibit a diverse array of interactions with their green hosts, ranging from beneficial to pathogenic, with the nature of these interactions contingent upon their phylogenetic position (Bonfante and Venice 2020). Conversely, Glomeromycota possess an ancient and ecologically significant symbiotic association with plants (Spatafora et al. 2016). Both Mucoromycota and Glomeromycota are considered to be among the earliest fungal lineages to have evolved specialized interactions with plants (Bonfante and Venice 2020).

## Conclusion

Plant development and nitrogen fertilization strongly influence the structure of rhizosphere associated the fungal microbiomes of Japonica rice, and substantially affect plant growth. The fungal community diversity was positively correlated with soil organic matter and microbial biomass. Throughout the growth of rice, the assembly of rice rhizosphere fungal community was mainly driven by the stochastic processes, and as the plant developed, the degree of assembly affected by deterministic factors increased. Glomeromycota, the most widespread AMF, was positively correlated with soil chemical properties and closely associated with soil nitrogen acquisition enzymes activities. However, the high nitrogen application rate adversely affected this fungi community in the rhizosphere of rice, which exhibited the highest abundance at the heading stage, suggesting that the interaction with the beneficial fungal community and soil biochemical properties might be an essential strategy for balancing the nitrogen demand and nitrogen fertilizer input. Our results illustrate how a shift in the fungal community mediates and reflect the effects of nitrogen fertilization input in rice agroecosystems. Improvement of soil fungal nitrogen metabolism processes to achieve higher nitrogen use efficiency and sustainable development should be considered in the future.

## Supplementary Information

**Additional file 1:** **Table S1.** Soil enzymes related to nitrogen metabolism and their functions. **Figure S1.** Diversity and richness of fungal communities in the rhizosphere. **Figure S2.** Relationship between soil fungal community composition (genus level) and soil biochemical properties.

## Data Availability

The raw FASTQ files obtained in the study for the sequencing libraries have been deposited into the NCBI Sequence Read Archive (SRA) under BioProject Accession Numbers PRJNA809892.

## References

[CR1] Averill C, Turner BL, Finzi AC (2014). Mycorrhiza-mediated competition between plants and decomposers drives soil carbon storage. Nature.

[CR2] Baldrian P, Valásková V (2008). Degradation of cellulose by basidiomycetous fungi. FEMS Microbiol Rev.

[CR3] Baldrian P, Kolařík M, Stursová M, Kopecký J, Valášková V, Větrovský T, Zifčáková L, Snajdr J, Rídl J, Vlček C, Voříšková J (2012). Active and total microbial communities in forest soil are largely different and highly stratified during decomposition. ISME J.

[CR4] Bao SD (2000). Soil agrochemical analysis.

[CR5] Berendsen RL, Pieterse CM, Bakker PA (2012). The rhizosphere microbiome and plant health. Trends Plant Sci.

[CR6] Bolyen E, Rideout JR, Dillon MR, Bokulich NA, Abnet CC, Al-Ghalith GA, Alexander H, Alm EJ, Arumugam M, Asnicar F, Bai Y, Bisanz JE, Bittinger K, Brejnrod A, Brislawn CJ, Brown CT, Callahan BJ, Caraballo-Rodríguez AM, Chase J, Cope EK, Da Silva R, Diener C, Dorrestein PC, Douglas GM, Durall DM, Duvallet C, Edwardson CF, Ernst M, Estaki M, Fouquier J, Gauglitz JM, Gibbons SM, Gibson DL, Gonzalez A, Gorlick K, Guo J, Hillmann B, Holmes S, Holste H, Huttenhower C, Huttley GA, Janssen S, Jarmusch AK, Jiang L, Kaehler BD, Kang KB, Keefe CR, Keim P, Kelley ST, Knights D, Koester I, Kosciolek T, Kreps J, Langille MGI, Lee J, Ley R, Liu Y-X, Loftfield E, Lozupone C, Maher M, Marotz C, Martin BD, McDonald D, McIver LJ, Melnik AV, Metcalf JL, Morgan SC, Morton JT, Naimey AT, Navas-Molina JA, Nothias LF, Orchanian SB, Pearson T, Peoples SL, Petras D, Preuss ML, Pruesse E, Rasmussen LB, Rivers A, Robeson MS, Rosenthal P, Segata N, Shaffer M, Shiffer A, Sinha R, Song SJ, Spear JR, Swafford AD, Thompson LR, Torres PJ, Trinh P, Tripathi A, Turnbaugh PJ, Ul-Hasan S, van der Hooft JJJ, Vargas F, Vázquez-Baeza Y, Vogtmann E, von Hippel M, Walters W, Wan Y, Wang M, Warren J, Weber KC, Williamson CHD, Willis AD, Xu ZZ, Zaneveld JR, Zhang Y, Zhu Q, Knight R, Caporaso JG (2019). Reproducible, interactive, scalable and extensible microbiome data science using QIIME 2. Nat Biotechnol.

[CR7] Bonfante P, Venice F (2020). Mucoromycota: going to the roots of plant-interacting fungi. Fungal Biol Rev.

[CR8] Breidenbach B, Pump J, Dumont MG (2015). Microbial community structure in the rhizosphere of rice plants. Front Microbiol.

[CR9] Chen Q-L, Hu H-W, Yan Z-Z, Li C-Y, Nguyen B-AT, Sun A-Q, Zhu Y-G, He J-Z (2021). Deterministic selection dominates microbial community assembly in termite mounds. Soil Biol Biochem.

[CR10] Dini-Andreote F, Stegen JC, van Elsas JD, Salles JF (2015). Disentangling mechanisms that mediate the balance between stochastic and deterministic processes in microbial succession. Proc Natl Acad Sci U S A.

[CR11] Dini-Andreote F, Stegen JC, van Elsas JD, Salles JF (2015). Disentangling mechanisms that mediate the balance between stochastic and deterministic processes in microbial succession. Proc Natl Acad Sci.

[CR12] Dong H, Fan S, Sun H, Chen C, Wang A, Jiang L, Ma D (2021). Rhizosphere-associated microbiomes of rice (Oryza sativa L.) under the effect of increased nitrogen fertilization [original research]. Front Microbiol.

[CR13] Edgar RC (2013). UPARSE: highly accurate OTU sequences from microbial amplicon reads. Nat Methods.

[CR14] Edwards J, Johnson C, Santos-Medellín C, Lurie E, Podishetty NK, Bhatnagar S, Eisen JA, Sundaresan V (2015). Structure, variation, and assembly of the root-associated microbiomes of rice. Proc Natl Acad Sci.

[CR15] Edwards JA, Santos-Medellín CM, Liechty ZS, Nguyen B, Lurie E, Eason S, Phillips G, Sundaresan V (2018). Compositional shifts in root-associated bacterial and archaeal microbiota track the plant life cycle in field-grown rice. PLoS Biol.

[CR16] Fan K, Weisenhorn P, Gilbert JA, Shi Y, Bai Y, Chu H (2018). Soil pH correlates with the co-occurrence and assemblage process of diazotrophic communities in rhizosphere and bulk soils of wheat fields. Soil Biol Biochem.

[CR17] Fan S, Zuo J, Dong H (2020). Changes in soil properties and bacterial community composition with biochar amendment after six years. Agronomy.

[CR18] Godfray HCJ, Beddington JR, Crute IR, Haddad L, Lawrence D, Muir JF, Pretty J, Robinson S, Thomas SM, Toulmin C (2010). Food security: the challenge of feeding 9 billion people. Science.

[CR19] Grinhut T, Hadar Y, Chen Y (2007). Degradation and transformation of humic substances by saprotrophic fungi: processes and mechanisms. Fungal Biol Rev.

[CR21] Jansson JK, Hofmockel KS (2018). The soil microbiome-from metagenomics to metaphenomics. Curr Opin Microbiol.

[CR22] Jiao S, Liu Z, Lin Y, Yang J, Chen W, Wei G (2016). Bacterial communities in oil contaminated soils: biogeography and co-occurrence patterns. Soil Biol Biochem.

[CR23] Jiao P, Li Z, Yang L, He J, Chang X, Xiao H, Nie X, Tong D (2021). Bacteria are more sensitive than fungi to moisture in eroded soil by natural grass vegetation restoration on the Loess Plateau. Sci Total Environ.

[CR24] Kennedy N, Connolly J, Clipson N (2005). Impact of lime, nitrogen and plant species on fungal community structure in grassland microcosms. Environ Microbiol.

[CR25] Kim H, Jeon J, Lee KK, Lee YH (2021). Compositional shift of bacterial, archaeal, and fungal communities is dependent on trophic lifestyles in rice paddy soil. Front Microbiol.

[CR26] Köhl L, Heijden M (2016). Arbuscular mycorrhizal fungal species differ in their effect on nutrient leaching. Soil Biol Biochem.

[CR27] Li S, Li Y, Hu C, Zheng X, Zhang J, Zhang H, Bai N, Zhang H, Tian M, Ban S (2021). Stochastic processes drive bacterial and fungal community assembly in sustainable intensive agricultural soils of Shanghai. China Sci Total Environ.

[CR28] Mendes LW, Kuramae EE, Navarrete AA, van Veen JA, Tsai SM (2014). Taxonomical and functional microbial community selection in soybean rhizosphere. ISME J.

[CR29] Nannipieri P, Trasar-Cepeda C, Dick RP (2017). Soil enzyme activity: a brief history and biochemistry as a basis for appropriate interpretations and meta-analysis. Biol Fertil Soils.

[CR30] Nguyen NH, Song Z, Bates ST, Branco S, Tedersoo L, Menke J, Schilling JS, Kennedy PG (2016). FUNGuild: an open annotation tool for parsing fungal community datasets by ecological guild. Fungal Ecol.

[CR31] Ofiteru ID, Lunn M, Curtis TP, Wells GF, Criddle CS, Francis CA, Sloan WT (2010). Combined niche and neutral effects in a microbial wastewater treatment community. Proc Natl Acad Sci U S A.

[CR32] Phillips RP, Brzostek E, Midgley MG (2013). The mycorrhizal-associated nutrient economy: a new framework for predicting carbon-nutrient couplings in temperate forests. New Phytol.

[CR33] Ruggiero P, Dec J, Bollag JM (1996). Soil as a catalytic system. Soil Biochem.

[CR34] Secilia J, Bagyaraj DJ (1994). Selection of efficient vesicular-arbuscular mycorrhizal fungi for wetland rice—a preliminary screen. Mycorrhiza.

[CR35] Solaiman MZ, Hirata H (1997). Effect of arbuscular mycorrhizal fungi inoculation of rice seedlings at the nursery stage upon performance in the paddy field and greenhouse. Plant Soil.

[CR36] Soong JL, Marañon-Jimenez S, Cotrufo MF, Boeckx P, Bodé S, Guenet B, Peñuelas J, Richter A, Stahl C, Verbruggen E (2018). Soil microbial CNP and respiration responses to organic matter and nutrient additions: evidence from a tropical soil incubation. Soil Biol Biochem.

[CR37] Spatafora JW, Ying C, Benny GL, Lazarus K, Stajich JE (2016). A phylum-level phylogenetic classification of zygomycete fungi based on genome-scale data. Mycologia.

[CR38] Stegen JC, Lin X, Konopka AE, Fredrickson JK (2012). Stochastic and deterministic assembly processes in subsurface microbial communities. ISME J.

[CR39] Stursová M, Zifčáková L, Leigh MB, Burgess R, Baldrian P (2012). Cellulose utilization in forest litter and soil: identification of bacterial and fungal decomposers. FEMS Microbiol Ecol.

[CR40] Tedersoo L, Naadel T, Bahram M, Pritsch K, Buegger F, Leal M, Kõljalg U, Põldmaa K (2012). Enzymatic activities and stable isotope patterns of ectomycorrhizal fungi in relation to phylogeny and exploration types in an afrotropical rain forest. New Phytol.

[CR41] Treseder KK, Lennon JT (2015). Fungal traits that drive ecosystem dynamics on land. Microbiol Mol Biol Rev.

[CR42] Xiao D, Che R, Liu X, Tan Y, Yang R, Zhang W, He X, Xu Z, Wang K (2019). Arbuscular mycorrhizal fungi abundance was sensitive to nitrogen addition but diversity was sensitive to phosphorus addition in karst ecosystems. Biol Fertil Soils.

[CR43] Zeng J, Liu X, Song L, Lin X, Zhang H, Shen C, Chu H (2016). Nitrogen fertilization directly affects soil bacterial diversity and indirectly affects bacterial community composition. Soil Biol Biochem.

[CR44] Zeng H, Yu L, Liu P, Wang Z, Chen Y, Wang J (2021). Nitrogen fertilization has a stronger influence than cropping pattern on AMF community in maize/soybean strip intercropping systems. Appl Soil Ecol.

[CR45] Zhalnina K, Louie KB, Hao Z, Mansoori N, da Rocha UN, Shi S, Cho H, Karaoz U, Loqué D, Bowen BP, Firestone MK, Northen TR, Brodie EL (2018). Dynamic root exudate chemistry and microbial substrate preferences drive patterns in rhizosphere microbial community assembly. Nat Microbiol.

[CR46] Zhang H, Sun Y, Xie X, Kim MS, Dowd SE, Paré PW (2009). A soil bacterium regulates plant acquisition of iron via deficiency-inducible mechanisms. Plant J.

[CR47] Zhang S, Wang L, Ma F, Bloomfield KJ, Yang J, Atkin OK (2014). Is resource allocation and grain yield of rice altered by inoculation with arbuscular mycorrhizal fungi?. J Plant Ecol.

[CR48] Zhang R, Vivanco JM, Shen Q (2017). The unseen rhizosphere root-soil-microbe interactions for crop production. Curr Opin Microbiol.

[CR49] Žifčáková L, Větrovský T, Howe A, Baldrian P (2016). Microbial activity in forest soil reflects the changes in ecosystem properties between summer and winter. Environ Microbiol.

