# Supplementary material for: Compositional Shifts and Assembly in Rhizosphere-Associated Fungal Microbiota Throughout the Life Cycle of Japonica Rice Under Increased Nitrogen Fertilization
Source: Rice (N Y). 2023 Aug 1;16:34. doi: 10.1186/s12284-023-00651-2 (PMC10393908; doi:10.1186/s12284-023-00651-2)
Supplement: Supplementary file 1 — Additional file 1: Table S1. Soil enzymes related to nitrogen metabolism and their functions. Figure S1. Diversity and richness of fungal communities in the rhizosphere. Figure S2. Relationship between soil fungal community composition (genus level) and soil biochemical properties. [file 12284_2023_651_MOESM1_ESM.docx]

**Supporting information**

**Table S1** Soil enzymes related to nitrogen metabolism and their functions.

| Soil enzyme | Abb. | Function |
| --- | --- | --- |
| Urease | UE | Hydrolyses urea to produce ammonia and carbonic acid (Fei et al., 2014) |
| Nitrate reductase | NR | Catalyses the reduction of the nitrate in the soil to nitrite (Morozkina & Zvyagilskaya, 2007) |
| Protease | PT | Transforms amino acids, proteins, and other organic compounds that contain protein nitrogen  (Jan et al., 2009) |
| Nitrogenase | NITS | Enzyme responsible for nitrogen fixation (Zhang et al., 2021) |
| 1,4-ß-N-acetylglucosaminidase | NAG | Hydrolyses oligomers of N-acetyl glucosamine (an amino sugar) in chitin (Moorhead et al., 2016) |
| Leucine aminopeptidase | LAP | Catalyses the cleavage of amino acids from proteins or other peptide substrates.(Moorhead et al., 2016) |

**Fig. S1** Diversity and richness of fungal communities in the rhizosphere subjected to three levels of nitrogen fertilization at the different growth stages, characterized by Shannon and Ace indices according to Alpha diversity analysis.

Different lowercase letters indicate significant difference between the nitrogen fertilization levels at the four growth stages at *p*<0.05 level,n=3.

a.

b.

**Fig. S2** Relationship between soil fungal community composition (genus level) and soil pH and nutrient content (a) and enzyme activity (b).

a. **
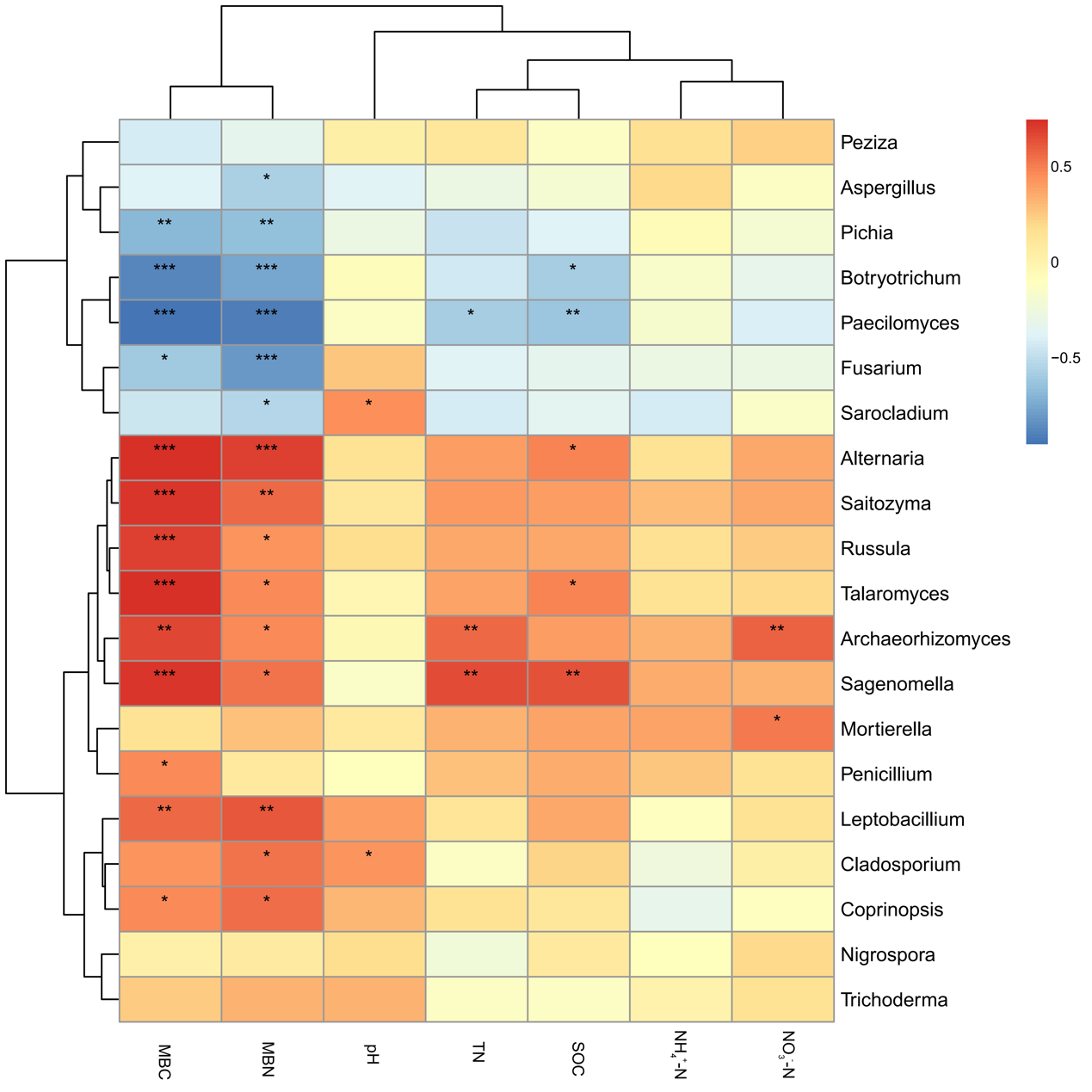
**

b.

**
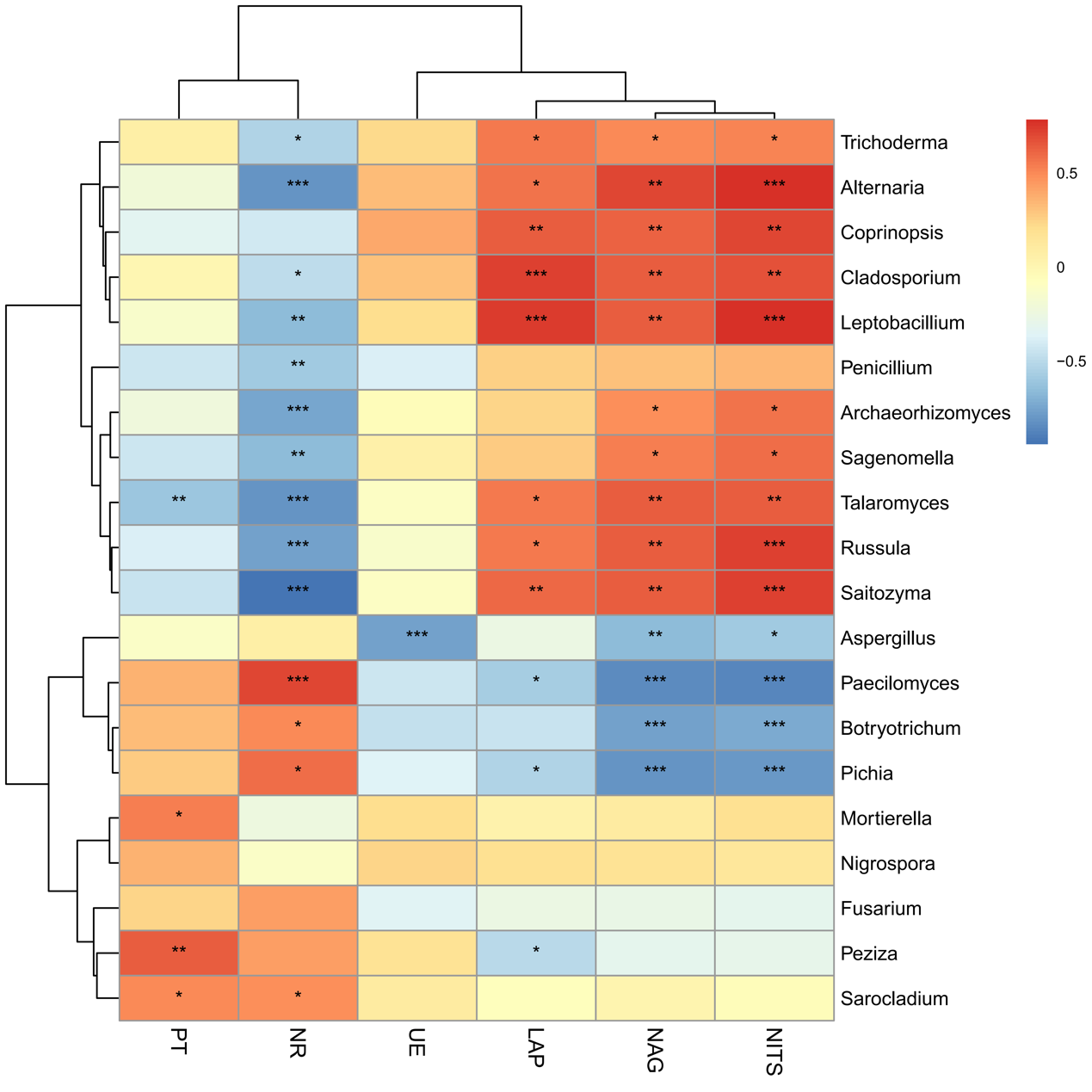
**

**References**

Fei, D., Wang, L., Ren, W. J., & Mei, X. F. (2014). Enhancing nitrogen utilization and soil nitrogen balance in paddy fields by optimizing nitrogen management and using polyaspartic acid urea. *Field Crops Research*, *169*, 30-38.

Morozkina, E. V., & Zvyagilskaya, R. A. (2007). Nitrate reductases: Structure, functions, and effect of stress factors. *Biochemistry (Moscow)*, *72*(10), 1151-1160. <https://doi.org/10.1134/S0006297907100124>

Jan, M. T., Roberts, P., Tonheim, S. K., & Jones, D. L. (2009). Protein breakdown represents a major bottleneck in nitrogen cycling in grassland soils. *Soil Biology and Biochemistry*, *41*(11), 2272-2282. [https://doi.org/https://doi.org/10.1016/j.soilbio.2009.08.013](https://doi.org/https:/doi.org/10.1016/j.soilbio.2009.08.013)

Zhang, J.-h., Huang, J., Hussain, S., Zhu, L.-f., Cao, X.-c., Zhu, C.-q., Jin, Q.-y., & Zhang, H. (2021). Increased ammonification, nitrogenase, soil respiration and microbial biomass N in the rhizosphere of rice plants inoculated with rhizobacteria. *Journal of Integrative Agriculture*, *20*(10), 2781-2796. [https://doi.org/https://doi.org/10.1016/S2095-3119(20)63454-2](https://doi.org/https:/doi.org/10.1016/S2095-3119(20)63454-2)

Moorhead, D. L., Sinsabaugh, R. L., Hill, B. H., & Weintraub, M. N. (2016). Vector analysis of ecoenzyme activities reveal constraints on coupled C, N and P dynamics. *Soil Biology and Biochemistry*, *93*, 1-7. [https://doi.org/https://doi.org/10.1016/j.soilbio.2015.10.019](https://doi.org/https:/doi.org/10.1016/j.soilbio.2015.10.019)
